# Case Report: Isolated diffuse alveolar hemorrhage as the sole severe manifestation of atypical leptospirosis without jaundice or acute kidney injury and the diagnostic value of BALF metagenomic next-generation sequencing

**DOI:** 10.3389/fmed.2026.1906337

**Published:** 2026-08-13

**Authors:** Jianxiong Xiao, Yina Wu, Zhixian Ding

**Affiliations:** 1 Intensive Care Unit, Wanbei Coal Electric Group General Hospital, Suzhou, Anhui, China; 2 Reproductive Medicine, Wanbei Coal Electric Group General Hospital, Suzhou, Anhui, China

**Keywords:** acute respiratory distress syndrome, diffuse alveolar hemorrhage, Leptospira spp., leptospirosis, metagenomic next-generation sequencing, zoonoses

## Abstract

**Background:**

Leptospirosis is a global zoonotic infection. Severe leptospirosis typically manifests as Weil’s syndrome with jaundice and acute kidney injury, while isolated diffuse alveolar hemorrhage (DAH) without jaundice or acute kidney injury (the classic features of Weil’s syndrome) is rare and easily misdiagnosed. Traditional diagnostic methods have notable limitations in the acute phase of infection, and the diagnostic value of metagenomic next-generation sequencing (mNGS) for atypical leptospirosis remains insufficiently characterized. This report highlights the clinical novelty of this rare phenotype and the diagnostic value of mNGS.

**Case presentation:**

A 60-year-old male farmer was admitted with 6 days of fever and 2 days of progressive chest tightness, with a history of contaminated water exposure via broken skin before symptom onset. Initial chest computed tomography showed bilateral pulmonary infiltrates, and he was misdiagnosed with severe community-acquired pneumonia and acute respiratory failure. Despite empiric broad-spectrum anti-infective therapy, his condition progressed rapidly, requiring invasive mechanical ventilation. Bronchoalveolar lavage fluid mNGS identified *Leptospira* spp., and the diagnosis was revised to leptospirosis with isolated DAH. After targeted anti-infective therapy combined with methylprednisolone pulse therapy, his respiratory function improved rapidly. He was weaned from mechanical ventilation on day 7 of admission, discharged with complete symptom resolution on day 19, and had no sequelae at 25-day follow-up.

**Conclusion:**

This case highlights that leptospirosis can present with isolated DAH as the sole severe manifestation without classic hepatorenal impairment, requiring high clinical suspicion in endemic areas. Bronchoalveolar lavage fluid mNGS may be a valuable tool for rapid diagnosis of atypical leptospirosis, which may reduce misdiagnosis and contribute to improved clinical outcomes.

## Introduction

Leptospirosis, a worldwide zoonotic infection caused by pathogenic spirochetes of the genus *Leptospira*, remains a major public health burden, especially in tropical and subtropical regions with high exposure to contaminated water or soil (1, 2). The clinical manifestations of leptospirosis are highly heterogeneous, ranging from mild flu-like symptoms to fatal severe disease. Classic severe leptospirosis, known as Weil’s syndrome, is characterized by the triad of fever, jaundice, and acute kidney injury (AKI), with a mortality rate of up to 40% (3, 4).

DAH is one of the most life-threatening complications of severe leptospirosis, with mortality exceeding 50% in patients with acute respiratory distress syndrome (ARDS) (5, 6). Previous studies have shown that leptospirosis-related DAH mostly occurs in patients with concurrent Weil’s syndrome, accompanied by multi-organ dysfunction (7, 8). However, isolated DAH without jaundice or hepatorenal impairment is a rare atypical presentation, which lacks specific clinical and imaging features, and is easily misdiagnosed as severe community-acquired pneumonia, influenza, or other acute respiratory infections (9). This misdiagnosis often leads to delayed targeted treatment and poor clinical outcomes.

The diagnosis of leptospirosis remains challenging in clinical practice. The gold standard blood culture has a low positive rate and requires a long incubation period, while serological tests such as microscopic agglutination test (MAT) have limited sensitivity in the acute phase of infection (10). Metagenomic next-generation sequencing (mNGS) has emerged as a powerful tool for the rapid detection of rare and difficult-to-culture pathogens, with the advantage of not being affected by prior antibiotic use (11, 12). However, clinical evidence on the application of BALF-mNGS in the diagnosis of atypical leptospirosis with isolated DAH is still limited.

Herein, we report a rare case of severe leptospirosis presenting with isolated DAH as the sole severe manifestation, without jaundice or hepatorenal dysfunction. The patient was rapidly diagnosed using BALF-mNGS, and achieved a favorable outcome after targeted anti-infective and glucocorticoid therapy. This case aims to raise clinical awareness of atypical leptospirosis and highlight the potential value of BALF-mNGS in early diagnosis, while acknowledging the need for confirmatory testing and cautious interpretation of low-read mNGS results.

### Case presentation

This case report was written in accordance with the CARE Guidelines for case reports.

A 60-year-old male farmer was admitted to the Respiratory and Critical Care Department of Wanbei Coal Electric Group General Hospital on August 19, 2021, with a 6-day history of fever and 2-day progressive chest tightness and dyspnea. The patient had no significant past medical history, no history of chronic cardiopulmonary disease, and no history of smoking or alcohol consumption. He had a history of outdoor fishing in contaminated water with broken skin on his extremities 3 days before the onset of symptoms, which was a key epidemiological exposure for leptospirosis.

On August 13, 2021, the patient developed fever after catching a cold, with a maximum body temperature of 38.6 °C, accompanied by intermittent cough and mild fatigue. The fever was irregular, and did not respond to oral antipyretic drugs at home. On August 17, 2021, he was admitted to the local county hospital. Initial blood routine examination showed white blood cell (WBC) count of 10.67 × 10^9^/L (reference range 3.5–9.5 × 10^9^/L), neutrophil percentage of 94.0%, lymphocyte percentage of 3.0%. Respiratory pathogen panel performed on nasopharyngeal swabs (covering *Legionella pneumophila*, *Mycoplasma pneumoniae*, *Chlamydia pneumoniae*, influenza A/B virus, respiratory syncytial virus, and adenovirus) was all negative. Chest computed tomography (CT) revealed bilateral pulmonary infectious infiltrates. He was empirically treated with broad-spectrum antibiotics, antipyretics and symptomatic support, but his symptoms progressively worsened. Repeat chest CT on August 18 showed significant progression of bilateral pulmonary lesions. For further treatment, he was transferred to our hospital on August 19, 2021.

### Vital signs and physical examination on admission

The patient was conscious, with a body temperature of 37.9 °C, heart rate of 120 beats per minute, respiratory rate of 22 breaths per minute, blood pressure of 112/75 mmHg, and oxygen saturation of 88% on room air. No skin rash, jaundice, or petechiae was observed on the whole body. No scleral icterus was present. Auscultation of the lungs revealed coarse breath sounds and bilateral moist crackles, without wheezing. Heart rhythm was regular, with no pathological murmurs. Abdominal examination was unremarkable, with no tenderness, rebound tenderness, or hepatosplenomegaly. No percussion tenderness in the liver or kidney areas was noted. No edema was observed in the lower extremities.

*Initial admission diagnosis*: 1. Severe community-acquired pneumonia; 2. Type I respiratory failure.

### Initial treatment and disease progression

After admission, the patient was given high-flow nasal cannula (HFNC) oxygen therapy (FiO₂ 45%, flow 50 L/min), empiric anti-infective therapy with meropenem (1 g q8h, intravenous) to cover potential pathogens causing severe community-acquired pneumonia, including gram-negative bacilli and anaerobes, in accordance with the Surviving Sepsis Campaign guidelines. Oseltamivir (75 mg q12h, oral) combined with acyclovir were administered for broad-spectrum antiviral coverage, as influenza and other viral pneumonias are common causes of acute respiratory failure during the rainy season in this region. Autoimmune workup including ANA, ANCA, anti-GBM antibodies was not performed immediately on admission. This decision was based on: (1) The patient’s clear epidemiological history of zoonotic exposure; (2) Absence of extrapulmonary manifestations suggestive of autoimmune disease (rash, arthritis, renal involvement); (3) Rapid clinical improvement after targeted antimicrobial and corticosteroid therapy, which made autoimmune etiologies less likely.

**Figure 1:**
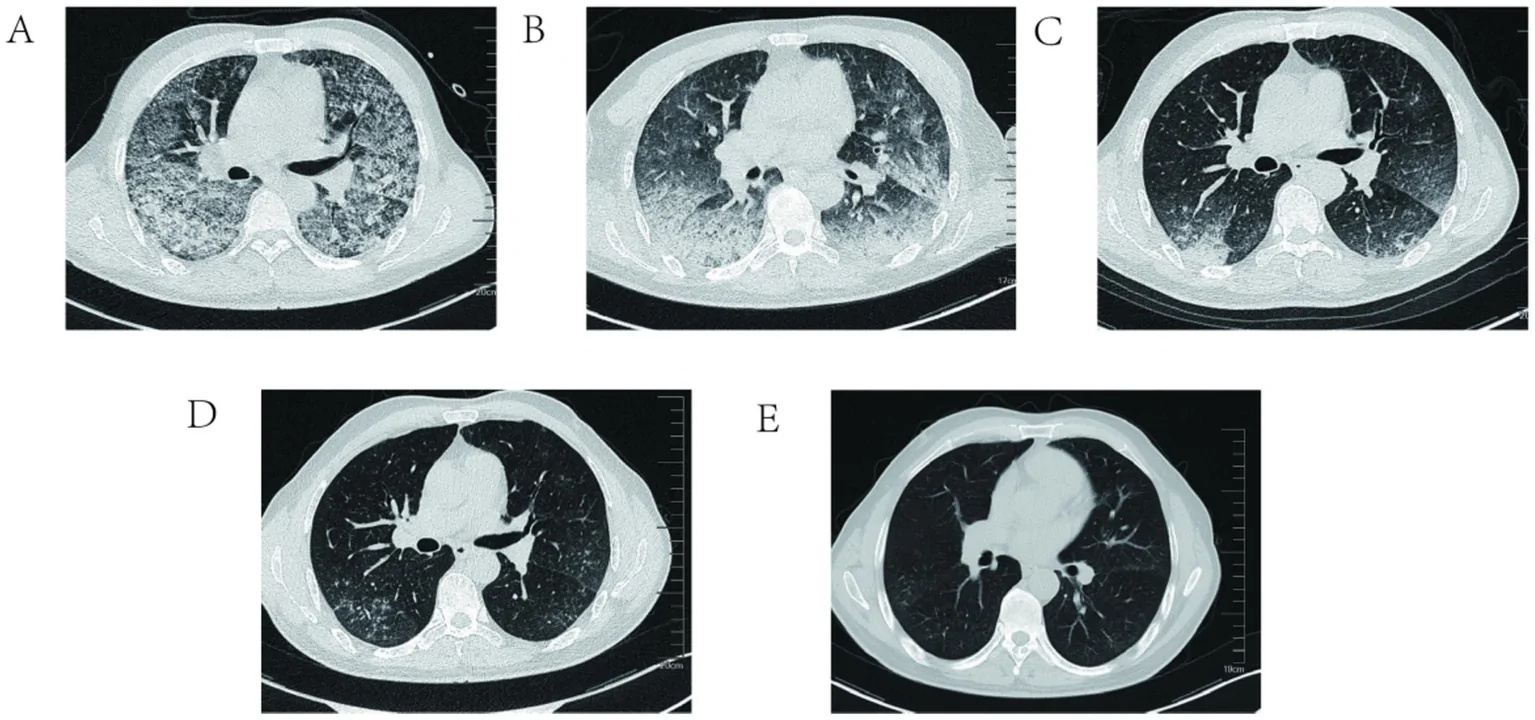
**(A)** Chest CT obtained on August 20, 2021, showing extensive patchy high-density shadows with diffuse ground-glass opacities in bilateral lungs, consistent with imaging manifestations of diffuse alveolar hemorrhage. **(B)** Chest CT obtained on August 25, 2021, showing partial absorption of bilateral pulmonary infiltrates. **(C)** Chest CT obtained on August 30, 2021, showing significant absorption of bilateral pulmonary lesions. **(D)** Chest CT obtained on September 6, 2021, showing further absorption of lesions with residual consolidation. **(E)** Chest CT obtained on October 2, 2021, showing almost complete absorption of pulmonary lesions without obvious fibrosis or sequelae.

In the early morning of August 20, 2021, the patient developed aggravated chest tightness, dyspnea, and hemoptysis, with oxygen saturation dropping to 82% under HFNC. He was immediately transferred to the intensive care unit (ICU). Repeat chest CT showed extensive patchy and flocculent high-density shadows in both lungs (Figure 1A). Laboratory tests on August 20 revealed anemia (hemoglobin 75 g/L), hypoalbuminemia (albumin 25.6 g/L), platelet count at the lower limit of normal (120 × 10^9^/L), and elevated inflammatory markers (C-reactive protein 135.86 mg/L). Notably, his serum creatinine remained within normal limits throughout hospitalization. Mild elevations in transaminases and transient hyperbilirubinemia were observed later during the course of illness (Table 1), but there was no evidence of severe liver dysfunction (total bilirubin >3 × upper limit of normal) or acute kidney injury, which are the hallmark features of classic Weil syndrome.

**Table 1:** Clinical course timeline and serial laboratory results.

Category	Test item (reference range)	20/8/21	21/8/21	23/8/21	26/8/21	28/8/21	30/8/21	4/9/21
Inflammation and hematology	C-reactive protein, CRP (0–10 mg/L)	135.86	147.86	93.08	20.79	/	4.90	1.40
	White blood cell count, WBC (3.5–9.5 × 10^9^/L)	15.4	23.89	16.16	13.09	10.92	11.79	6.29
	Hemoglobin, HB (120–160 g/L)	75	52	86	79	93	94	87
	Platelet count, PLT (100–300 × 10^9^/L)	120	176	196	237	274	238	221
	Albumin (35–55 g/L)	25.6	/	33.8	31.9	34.0	32.2	33.2
Liver and kidney function	Creatinine (57–111 μmol/L)	89	/	67	48	/	/	44
	Total Bilirubin (3.4–17.1 μmol/L)	17.0	/	20.8	35.0	51.0	37.8	22.0
	Aspartate aminotransferase, AST (0–40 U/L)	85	/	36	48	31	37	30
	Alanine aminotransferase, ALT (0–40 U/L)	57	/	32	88	70	42	40
Blood gas and respiratory support	Fraction of inspired oxygen, FiO₂	45% (HFNC)	45% (PCV)	45% (PCV)	40% (PCV)	35% (HFNC)	30% (HFNC)	/
	Positive end-expiratory pressure, PEEP	/	10	8	6	/	/	/
	pH (7.35–7.45)	7.46	7.38	7.35	7.54	7.49	7.44	/
	Partial pressure of oxygen, PaO₂ (80–100 mmHg)	74	55	101	107	79	86	/
	PaO₂/FiO₂ ratio (>300 mmHg)	164	122	224	268	226	287	/
	Partial pressure of carbon dioxide, PaCO₂ (35–45 mmHg)	26	44	50	30	29	33	/
	Lactic acid (0.5–1.6 mmol/L)	0.7	1.6	1.0	0.8	0.7	0.8	/

“/” indicates no test was performed at this time point; HFNC, High-flow nasal cannula; PCV, Pressure-controlled ventilation.

On the afternoon of August 20, the patient’s respiratory failure continued to worsen, with a PaO2/FiO2 ratio of 186 mmHg (meeting the Berlin definition of moderate ARDS). Endotracheal intubation and invasive mechanical ventilation were immediately performed, with a lung-protective ventilation strategy: pressure-controlled ventilation, tidal volume 6 mL/kg predicted body weight, plateau pressure <30 cmH₂O, driving pressure <15 cmH₂O, and PEEP 10 cmH₂O titrated to maintain oxygen saturation >92%. Meanwhile, fiberoptic bronchoscopy was performed, which showed a moderate amount of diffuse bloody secretions in the bilateral bronchial tree. Due to the patient’s critical condition (hypoxemia requiring high PEEP mechanical ventilation) and unstable hemodynamics, sequential bronchoalveolar lavage and cytological examination for hemosiderin-laden macrophages (the gold standard for DAH diagnosis) could not be safely performed due to the patient’s critical condition and unstable hemodynamics. Furthermore, hemosiderin-laden macrophages typically require 24–72 h to appear after hemorrhage, which limited their utility for immediate diagnosis. However, the combination of diffuse bilateral bloody secretions on bronchoscopy, typical CT findings of diffuse ground-glass opacities, and rapidly progressive hypoxemic respiratory failure was sufficient for a clinical diagnosis of DAH, which is widely accepted in critical care settings. Bronchoalveolar lavage fluid (BALF) was collected and sent for mNGS testing, along with routine culture and pathogen panel.

### mNGS detection method

mNGS was performed on the BALF sample using the Illumina NovaSeq platform. A total of 20 million high-quality reads were generated after quality control and removal of human host sequences. The positivity threshold for fastidious pathogens such as Leptospira was set as ≥1 unique read, which is the standard threshold used in clinical microbiology laboratories. Sterile saline was processed in parallel as a negative control, and no Leptospira reads were detected in the control sample. The sequencing run generated approximately 20 million raw reads; after removal of human host sequences, approximately several million microbial reads remained for downstream analysis. The single Leptospira read corresponded to a very low genome coverage and relative abundance (less than 0.01% of total microbial reads). Species-level classification was not pursued due to the limited read depth. Pathogen identification was performed using the NCBI GenBank non-redundant database (version released August 2021).

### Etiological diagnosis and treatment adjustment

On August 22, 2021, the BALF-mNGS report identified 1 unique read mapping to *Leptospira* spp. (Table 2), while routine bacterial and fungal cultures of BALF showed no pathogenic growth after 7 days of standard incubation. Although only 1 read was detected, this finding was considered clinically significant because: (1) Leptospira is a fastidious spirochete that is difficult to culture, and single-read detection has been reported in mNGS for leptospirosis, particularly in samples with low bacterial burden; (2) The patient had a clear epidemiological history of contaminated water exposure with broken skin; (3) No other causative pathogens were identified after comprehensive screening; (4) The patient showed rapid clinical improvement only after targeted anti-leptospiral therapy. Combined with the patient’s epidemiological exposure history, clinical manifestations, and mNGS results, the diagnosis was revised to severe leptospirosis with isolated DAH, based on a comprehensive assessment of epidemiology, clinical presentation, and mNGS findings.

**Table 2:** Pathogen detection results of BALF metagenomic next-generation sequencing.

Genus	Species	Number of detected reads
*Leptospira*	Unidentified species (not distinguishable at this read depth)	1

Immediately after diagnosis, the anti-infective regimen was adjusted to intravenous ceftriaxone sodium (2 g q12h), which is the WHO-recommended first-line antibiotic for severe leptospirosis. Moxifloxacin (0.4 g qd) was added to provide additional coverage for potential secondary bacterial infections and to enhance anti-leptospiral activity, as fluoroquinolones have excellent *in vitro* activity against Leptospira and good lung tissue penetration. Methylprednisolone sodium succinate pulse therapy (500 mg qd, intravenous) was initiated for 3 consecutive days for the treatment of DAH-related inflammatory cascade and pulmonary vascular endothelial injury. The previous empiric meropenem, oseltamivir, and acyclovir were discontinued. Other supportive treatments included human serum albumin infusion to correct hypoalbuminemia, maintenance of fluid and electrolyte balance, and respiratory support optimization.

### Clinical course and outcome

After the adjustment of treatment, the patient’s respiratory function and inflammatory markers improved gradually. On August 21, the patient received 4 units of packed red blood cell transfusion due to a drop in hemoglobin to 52 g/L, which contributed to the subsequent rise to 86 g/L by August 23. On August 25, repeat chest CT showed that the bilateral pulmonary lesions were partially absorbed (Figure 1B). The dose of methylprednisolone was tapered to 40 mg q12h. The total duration of methylprednisolone pulse therapy (500 mg daily) was 3 days, followed by a taper over 7 days, and the antibiotic course (ceftriaxone plus moxifloxacin) was continued for a total of 14 days. As the patient’s oxygenation, inflammatory markers and imaging lesions all showed significant improvement, to avoid adverse effects of long-term high-dose glucocorticoid. On August 26, the patient’s PaO_2_/FiO_2_ ratio increased to 268 mmHg, with clear consciousness, satisfactory cough reflex, and stable hemodynamics. He was successfully weaned from mechanical ventilation and extubated, and switched to HFNC oxygen therapy.

On August 30, the patient’s chest tightness and hemoptysis resolved completely, with significant absorption of pulmonary lesions on chest CT (Figure 1C). He was transferred to the general respiratory ward for sequential treatment. On September 6, chest CT showed further absorption of pulmonary lesions with partial consolidation (Figure 1D). The patient’s vital signs were stable, laboratory tests returned to normal (Table 1), and he was discharged on September 7, 2021 (19 days after admission).

### Follow-up

A detailed clinical timeline is provided in the Supplementary Material. The patient was followed up via telephone on October 7, 2021 (30 days after discharge), with no residual respiratory symptoms or discomfort. Outpatient chest CT re-examination on October 2, 2021 (25 days after discharge) showed almost complete absorption of bilateral pulmonary lesions, with no obvious fibrosis or sequelae (Figure 1E). The patient expressed gratitude for the timely diagnosis and treatment, and stated that he had fully recovered and returned to normal agricultural work.

Figure1 A Chest CT obtained on August 20, 2021 (before invasive mechanical ventilation): Extensive patchy and flocculent high-density shadows with diffuse ground-glass opacities in bilateral lungs, consistent with imaging manifestations of diffuse alveolar hemorrhage Figure 1B. Chest CT obtained on August 25, 2021 (3 days after targeted anti-infective and glucocorticoid pulse therapy): Partial absorption of bilateral pulmonary infiltrates, with reduced range of high-density shadows and ground-glass opacities Figure 1C. Chest CT obtained on August 30, 2021 (4 days after weaning from mechanical ventilation): Significant absorption of bilateral pulmonary lesions, with markedly decreased range of consolidation and exudation Figure 1D. Chest CT obtained on September 6, 2021 (1 day before discharge): Further absorption of pulmonary infectious lesions, with residual partial consolidation and no new exudative lesions Figure 1E. Chest CT obtained on October 2, 2021 (25 days after discharge): Almost complete absorption of bilateral pulmonary lesions, no obvious pulmonary fibrosis or long-term sequelae.

## Discussion

In this case report, we described a rare case of severe leptospirosis presenting with isolated DAH as the sole severe manifestation, without the classic jaundice and severe hepatorenal impairment (Weil’s syndrome). The patient was initially misdiagnosed with severe community-acquired pneumonia and ARDS, and was rapidly diagnosed by BALF-mNGS, which contributed to a favorable outcome after targeted anti-infective and glucocorticoid pulse therapy. It is worth noting that the patient showed persistent clinical deterioration during meropenem therapy, despite its partial *in vitro* activity against Leptospira. Rapid improvement was only observed after switching to ceftriaxone and initiating high-dose corticosteroid treatment, suggesting that targeted anti-leptospiral therapy and immunomodulation were the key contributors to recovery.

The first key clinical highlight of this case is the uncommon atypical presentation of leptospirosis. Classic severe leptospirosis (Weil’s syndrome) is characterized by fever, jaundice, and AKI, and DAH mostly occurs in patients with concurrent multi-organ dysfunction (13, 14). Although leptospirosis-related DAH frequently occurs in patients with concurrent Weil’s syndrome and multi-organ dysfunction, isolated DAH without severe hepatorenal involvement has been only sporadically documented in the literature (9, 15). Consistent with these reports, our patient had no jaundice and did not develop acute kidney injury. Although transient mild elevations in transaminases and bilirubin (peak total bilirubin 51.0 μmol/L) were observed, these did not meet the criteria for severe hepatic failure or Weil’s syndrome, and DAH was the only severe and life-threatening manifestation. This atypical presentation is the main cause of misdiagnosis, as it lacks the classic clinical features that prompt clinicians to consider leptospirosis. In this case, the patient was initially treated with broad-spectrum antibiotics and antiviral drugs, but his condition continued to deteriorate, which further highlights the need for high clinical suspicion of leptospirosis in patients with unexplained progressive DAH, even in the absence of hepatorenal impairment, especially in endemic areas with a history of water exposure.

The second key highlight of this case is the diagnostic value of BALF-mNGS for atypical leptospirosis. The early diagnosis of leptospirosis remains a major clinical challenge. The gold standard blood culture has a positive rate of less than 50% and requires 2–6 weeks of incubation, which is not suitable for early diagnosis in critically ill patients (16, 17). Serological tests such as MAT are the most commonly used diagnostic method, but antibodies usually appear 7–10 days after the onset of symptoms, and the sensitivity is less than 40% in the first week of illness (18, 19). In this case, the patient was admitted on the 6th day of illness, which was in the acute phase of infection, and serological tests may have false negative results.

In recent years, mNGS has been increasingly used in the diagnosis of severe infectious diseases, with the advantages of rapid detection, high sensitivity, and no need for prior pathogen prediction (20, 21). Unlike traditional culture methods, mNGS is not affected by prior antibiotic use, which is particularly important for critically ill patients who have already received empiric antibiotic treatment (22). In 2014, Wilson et al. first reported the use of cerebrospinal fluid mNGS to diagnose neuroleptospirosis, which confirmed the value of mNGS in the diagnosis of leptospirosis (23, 24). However, most previous studies focused on blood or cerebrospinal fluid samples, and clinical evidence on BALF-mNGS for leptospirosis-related DAH is still limited. In this case, BALF-mNGS identified *Leptospira* spp. within 48 h, which directly guided the adjustment of treatment and was associated with subsequent clinical improvement. This case further suggests that BALF-mNGS is a valuable tool for the early diagnosis of atypical leptospirosis with pulmonary manifestations, especially for patients with unexplained DAH who have received empiric antibiotic treatment (25–27). Notably, only 1 specific read of *Leptospira* spp. was detected in BALF-mNGS in this case, but the diagnosis was strongly supported by three lines of evidence: first, the patient had a clear high-risk epidemiological exposure history—outdoor fishing in contaminated water with broken skin, which is the classic transmission route of leptospirosis; second, no other causative pathogens were identified after comprehensive pathogen screening, including respiratory pathogen panel, routine bacterial/fungal cultures of BALF; third, the patient’s respiratory symptoms, inflammatory markers and imaging lesions all improved rapidly and significantly after targeted *anti-leptospira* therapy, which provided compelling clinical support for the mNGS finding. Previous studies have also confirmed that the diagnosis of pathogens by mNGS should be comprehensively judged in combination with clinical characteristics, rather than simply relying on the number of detected reads, especially for fastidious pathogens like *Leptospira* that are difficult to culture.

In terms of treatment, our patient achieved a favorable outcome after targeted anti-infective therapy combined with glucocorticoid pulse therapy. Ceftriaxone is the first-line antibiotic for severe leptospirosis, which can effectively clear *Leptospira* and reduce the duration of disease (28). The combination of moxifloxacin can enhance the bactericidal effect, especially for severe cases. Notably, the role of glucocorticoids in leptospirosis-related DAH remains controversial in clinical practice. Some randomized controlled trials have not found a significant mortality benefit of routine glucocorticoid use in severe leptospirosis, but a large number of real-world studies and case series support that high-dose pulse therapy can effectively inhibit the cytokine storm and vascular endothelial injury caused by *Leptospira* infection, thereby reducing pulmonary hemorrhage and improving oxygenation in DAH patients (29, 30). In this case, the patient’s respiratory function improved rapidly after methylprednisolone pulse therapy, which suggests that glucocorticoid pulse therapy may be a useful adjunctive treatment, although the favorable outcome in this case was temporally associated with multiple concurrent interventions (ceftriaxone, moxifloxacin, corticosteroids, and supportive care), and cannot be attributed to any single agent, even in patients without multi-organ dysfunction.

This case has several limitations. First, it is a single case report, and the generalizability of the findings is limited. Second, we did not perform PCR or paired microscopic agglutination testing (MAT) for confirmation of leptospirosis, as no stored specimens were available for retrospective testing; this limits the diagnostic certainty. Third, we did not perform sequential Broncho alveolar lavage to detect hemosiderin-laden macrophages, which was not feasible due to the patient’s critical condition. Fourth, we did not explore the specific pathophysiological mechanism of isolated DAH in this case, which needs further basic and clinical research.

In summary, this rare case highlights that leptospirosis can present with isolated DAH as the sole severe manifestation, without the classic features of Weil’s syndrome, which is prone to misdiagnosis. BALF-mNGS can achieve rapid diagnosis of atypical leptospirosis, which is crucial for improving clinical outcomes. Clinicians in endemic areas should maintain high suspicion of leptospirosis in patients with unexplained DAH, even in the absence of hepatorenal impairment, and mNGS should be performed as early as possible to confirm the diagnosis.

## Conclusion

This rare case highlights that leptospirosis can present with isolated DAH as the sole life-threatening manifestation, even in the absence of classic jaundice and severe hepatorenal failure of Weil’s syndrome. This atypical phenotype is highly prone to misdiagnosis as severe community-acquired pneumonia, leading to delayed targeted treatment and poor prognosis. For patients in endemic areas with unexplained progressive DAH and a history of contaminated water exposure, clinicians should maintain high clinical suspicion of leptospirosis, even without hepatorenal dysfunction. BALF-mNGS may be a valuable tool for rapid etiological diagnosis of such atypical cases, especially for patients who have received empiric antibiotic treatment. Early initiation of targeted anti-leptospiral therapy combined with glucocorticoid pulse therapy may effectively improve the prognosis of patients with leptospirosis-related DAH. Further large-scale studies are needed to confirm these findings.

## Data Availability

The original contributions presented in the study are included in the article/supplementary material, further inquiries can be directed to the corresponding author.
